# An Initial Investigation of the Generalization of a School-Based Social Competence Intervention for Youth with High-Functioning Autism

**DOI:** 10.1155/2011/589539

**Published:** 2011-11-16

**Authors:** Carla Schmidt, Janine P. Stichter, Kristin Lierheimer, Stephanie McGhee, Karen V. O'Connor

**Affiliations:** ^1^Juniper Gardens Children's Project, The University of Kansas, Kansas City, KS 66101, USA; ^2^Department of Special Education, The University of Missouri, Columbia, MO 65211, USA

## Abstract

This study evaluated the impact of generalization of the Social Competence Intervention-Adolescent (SCI-A) curriculum in a school setting for individuals with high-functioning autism or Asperger's Syndrome (*N* = 6). This study examined to what degree the generalization of the SCI-A curriculum could be measured when delivered in a school setting. Across the six participants preliminary results suggest improvement on teacher reports of social skills and executive functioning. Some improvements were also evident in direct measures of facial-expression recognition. Data collected in the nonintervention settings indicated that some generalization of social interaction skills may have occurred for all six participants. Future research directions are discussed.

## 1. Introduction

Autism Spectrum Disorders (ASDs) are pervasive developmental disorders that have lasting impact on social interaction and communication skills, greatly influencing an individual's independent functioning, and quality of life. According to the Diagnostic and Statistical Manual of Mental Disorders—4th Edition-Text Revision* (DSM-IV-TR)*; [1], Pervasive Developmental Disorders (PDDs) are all characterized by “severe and pervasive impairments in several areas of development: reciprocal social interaction skills, communication skills, or the presence of stereotyped behaviors, interests, and activities” (page 69). Asperger's Syndrome (AS) or high-functioning autism (HFA) is part of the continuum of disorders classified as PDDs; yet individuals classified with these disorders tend to exhibit milder autism symptomology compared to other disorders on the spectrum. Nevertheless these individuals remain greatly impacted by deficits in social competence.

Deficits in social competence, if unremediated, can lead to a number of negative outcomes for adults with HFA/AS. Although these individuals have intellectual functioning within the normal range (IQ 70+) and often excel in academic subjects [2], deficits in social functioning can prevent these individuals from achieving full independence into adulthood [3]. Long-term outcome studies for individuals with HFA/AS report persisting impairments in adaptive and social functioning as well as psychiatric disorders throughout adulthood [4, 5]. Additionally, individuals with HFA/AS remain reliant on families or community services into adulthood. In spite of obtaining high school, and even college diplomas, these individuals have difficulty finding and maintaining employment [4]. Meaningful relationships are an additional challenge for this population with a number of participants never developing relationships outside of their families. Howlin [6] note that due to these variables, individuals with HFA/AS are at risk for frustration, loss of self-esteem, anxiety, and severe depression. Therefore the literature proposes that intervention goals should focus on social behaviors that allow individuals with HFA/AS to improve the depth and quality of their social relationships, in order to achieve satisfying, supportive, and meaningful relationships [4, 5, 7]. It is hypothesized that an increase in these relationships will have positive and long-lasting impact across multiple domains [4]. 

Unfortunately to date there are insufficient evidence-based social interventions for individuals with HFA/AS [8, 9]. In fact, The National Autism Center (NAC) recently published the *National Standards Report* [10], an extensive evaluation of current evidence-based practices for ASD. According to this report, a total of 11 treatments were identified as established treatments and 22 as emerging. Interestingly, of the established treatments, only two reported evidence for individuals with HFA/AS and for the emerging treatments only four treatments reported evidence for HFA/AS, therefore, highlighting the need for more research to validate social and behavioral interventions to support the specific deficit areas of individuals with HFA/AS. 

The field has also called attention to the one persistent challenge, to develop and validate interventions that promote generalization and maintenance of intervention skills [11]. Although the challenge has been recognized, according to recent literature reviews, few studies to date report generalization data. In a review of group-based social skills training programs for adolescents, White et al. [12] concluded that generalization and flexibility of skill use in natural environments continues to be a challenge and that the lack of investigation of the degree to which skills generalize is a major methodological weakness in the social competence literature. In their review of early social communicative skills of young children with ASD, Hwang and Hughes [13] reported that only 9 of the 16 articles included reported generalization data. Rao et al. [9] reviewed the social skills training literature for individuals with HFA/AS and only three of the ten studies reported generalization data. In conclusion, unless the social skills acquired through intervention can generalize to novel settings with novel individuals, the potential and demonstrated efficacy of social competence interventions for individuals with HFA/AS remains limited [14].

One category of social competence intervention that is beginning to report positive social and behavioral outcomes for individuals with HFA/AS is based on a cognitive behavioral intervention (CBI) framework [15–17]. CBI is considered one of the emerging practices by the NAC [10] for individuals with HFA/AS.

The success of interventions using a CBI framework with this population may be due to the focus on constructs such as theory of mind, emotion recognition, and executive functioning, as it is these three core social competence constructs that have been suggested by the research literature to contribute to the unique social deficits of individuals with HFA/AS (see [17]). Stichter and colleagues [17] have recently published encouraging findings for a social competence intervention (SCI) designed specifically to address these unique social deficits and is based on a CBI theoretical framework using applied behavior analysis principles. These authors reported results for 29 male participants with HFA/AS between the ages of 11 and 14. This group-based intervention was delivered in an after-school clinical setting for a total of 20-hour, meeting twice weekly, for ten weeks. A pre-post design using individually administered standardized assessments was used to examine the impact of participation in the SCI on overall social abilities, theory of mind, emotion recognition, and executive functioning. According to the results, the SCI curriculum increased social competence for all participants. Specifically, participants showed significant gains in overall social abilities, an increased ability to correctly identify emotions and mental states, as well as marked improvement in executive functioning skills. The authors reported a high degree of variability in participant's performance on the theory of mind measures and therefore no consistent pre-postimprovements were found. The results of Stichter et al. [17] were consistent with and extended previous similar work using CBI-based interventions (e.g., [15, 16]). 

Recently, guidelines have been developed to inform the design of research studies for psychosocial interventions for individuals with ASD [18]. The working group identified four phases of research: (1) formulation and systematic application of a new intervention technique, (2) developing a manual and research plan for evaluation of the intervention across sites, (3) randomized clinical trials, and (4) community effectiveness studies [18]. Stichter et al. [17] represents the first phase of research outlined by Smith and colleagues [18]. The current study represents one of several initial evaluations of the second phase of research for this program.

The SCI-A curriculum was developed and implemented across seven groups of adolescents over five semesters in an after-school, clinic-based program. The SCI research team secured federal funding to further develop the SCI curriculum, translate the clinic-based curriculum to a school-based delivery, and implement this curriculum across multiple research sites. Therefore, the purpose of this study is to (1) replicate and extend the work of Stichter and colleagues [17] by conducting a preliminary evaluation of the Social Competence Intervention-Adolescent (SCI-A) curriculum in a school setting and (2) to examine to what degree the generalization of the SCI-A curriculum could be measured when delivered in a school setting. A pre-post design was implemented in which the three core constructs of theory of mind, emotion recognition, and executive functioning were assessed using individually administered standardized assessments. In order to examine the generalization of the interaction skills taught within SCI-A to nonintervention settings, direct observation interactions of target students with adults and typically developing peers were measured before and throughout the SCI-A program.

## 2. Methods

### 2.1. Participants

All 6 participants were referred by teachers to project staff as having social deficits that would benefit from enhanced intervention, would be able to have their schedules adjusted to meet as a group at the same time, and met the inclusion criteria. Participants had confirmed ASD diagnosis by either the Autism Diagnostic Observation Schedule (ADOS); [19] or Autism Diagnostic Interview-Revised (ADI-R); [20], they were between the ages of 11 and 14, had a full-scale IQ of 75 or above, and participated in a general education setting for at least one hour a day. Records review was utilized for obtaining IQ scores. Recognizing the challenges and variations among the different intelligence/cognitive assessments and the divergent timing of these assessments, we attempted to gather the Wechsler Abbreviated Scale of Intelligence (WASI) [21] data whenever possible, approximately 2–4 weeks prior to the start of SCI-A instruction. All participants attended the same special education resource classroom in traditional middle school where they switched classes each period. Participants were receiving no other social skills instruction at the time of the current study. Specific information on inclusion criteria and subject characteristics are provided in Tables 1 and 2. 

### 2.2. The Social Competence Intervention-Adolescent

The SCI-A program was conducted in the schools to provide participants with a targeted social competence program specific to a subtype of individuals with ASD. Participants for the current study were recruited from within the SCI-A program. SCI-A is an extension of the original after-school model developed by Stichter and colleagues [17] and the current study was delivered within a midwestern middle school (6-7th grade). The intervention was delivered within a special education resource classroom by a specially trained site facilitator with a Master's degree and teaching license in special education and the licensed special education teacher who was scheduled to teach those students at that time. All participants attended the same special education resource classroom and met as a group for the SCI-A program. The five curricular units include (a) recognizing facial expressions, (b) sharing ideas, (c) turn taking in conversations, (d) recognizing feelings and emotions of self and others, and (e) problem solving. The project staff was responsible for all curricular content as designed and measures were taken to assure fidelity across interventionists.

### 2.3. Intervention and Generalization Settings

In total, SCI consisted of 20 hours of group intervention conducted twice a week for 10 weeks. SCI-A was delivered in five units (each comprised of four, one-hour lessons). Each of the five two-week units scaffolded learning, building upon each skill, with maintenance of learned skills reinforced throughout. The SCI-A program was delivered in a special education resource classroom during participant's regularly scheduled class time. In addition to the intervention setting, there were two generalization settings, lunch and math class, used in the current study. Generalization data were collected for all six participants in the lunch setting, and due to class schedule conflicts data were collected for only three of the participants in the math class. The lunch setting was chosen because this setting provides opportunity for naturally occurring social interaction with typically developing peers. The math setting was chosen because the curriculum used by the target Middle School by design affords weekly opportunities for group work and structured interaction. This structure was observed to provide natural opportunities within an academic setting for the use of social competence skills.

### 2.4. Measures

#### 2.4.1. Social Abilities

The Social Responsiveness Scale (SRS) [22] is a 65-item questionnaire designed for children or adolescents between the ages of 4 and 18 and was completed by teachers for each participant. The questionnaire focuses on the social symptoms of ASD by assessing five social areas and five treatment subscales. The social areas are social awareness, social information processing, capacity for reciprocal social communication, social anxiety/avoidance, and autistic preoccupation and traits. The five subscales are receptive aspects of social behavior, cognitive aspects of social behavior, expressive aspects of social behavior, motivational aspects of social behavior, and autistic mannerisms. A single score is generated that serves as an index of severity of social deficits across the autism spectrum. The SRS uses a Likert scale response format to assess symptom severity. Each item on the scale is rated using a range from 0 (never true) to 3 (almost always true). The SRS has shown to be a consistently reliable and valid measure [23–25]. 

#### 2.4.2. Theory of Mind

The SCI-A assessment battery includes five ToM tasks. Participants were given two first-order ToM tasks including the Sally-Anne false-belief task [26] and the Smarties false-belief task [27]. First-order ToM tasks require participants to attribute mental states to another person [28]. Participants were also given two second-order ToM tasks including the Friends ABC Story (adapted from [29]) and the Ice Cream Van Test [30]. Second-order ToM tasks require participants to predict one person's thoughts about another person's thoughts [28]. All of the ToM measures were scored on a pass/fail basis. Additionally, the Faux Pas Stories were administered [31]. This test consists of ten short narratives in which a social faux pas occurs. The Faux Pas Stories are scored by the number of correctly identified faux pas scenarios out of ten, with higher scores indicating a greater accuracy of faux pas identification [17].

#### 2.4.3. Emotion Recognition

Two measures were used to measure emotion recognition, the Diagnostic Analysis of Nonverbal Accuracy-2, Child Facial Expressions (DANVA 2-CF); [32], and the Reading the Mind in the Eyes test [33]. The DANVA 2-CF consists of 24 photographs of child facial expressions and is used to measure the ability to recognize facial expressions. The test includes an equal number of male (12) and female (12) faces and an equal distribution of high and low intensity, happy, sad, angry, and fearful faces. The DANVA 2-CF has been shown to be both a reliable and valid measure of facial expression processing [32]. The Reading the Mind in the Eyes consists of 28 photographs of the eye regions of the face of both male and females. The test is intended to measure how well participants can interpret the mental states of others based on reading facial expressions. Scores were calculated as the number of correct identifications, with higher scores indicating greater ability to interpret mental states based on facial cues.

#### 2.4.4. Executive Functioning

The Behavior Rating Inventory of Executive Function (BRIEF) [34] is an 86-item questionnaire designed to assess the behavioral manifestations of children's executive control functions in the home and school environments. Decreasing scores on the BRIEF indicate improvements in perceived executive functioning. The BRIEF is considered to be a reliable and valid measure of executive function [34].

### 2.5. Procedure

This study was conducted across the span of approximately fifteen weeks. Two different sets of data were collected for this study. The first data set included pre- and postassessment data for the SCI-A assessment battery administered by the SCI-A research team outlined above. Pre- and postassessment data were collected for all participants two weeks prior to and two weeks following the conclusion of the SCI-A program. The second data set included the direct observation of in situ social behavior (target behaviors) within the untrained settings before (baseline) and during the SCI-A program (intervention). Baseline data collection occurred for two weeks prior to the beginning of the SCI-A program and intervention data collection occurred for ten weeks during the implementation of the SCI-A program. The first, third, fourth, and fifth authors, who were knowledgeable of, but not directly involved in delivering the SCI-A program, collected all direct observation data. Consent and assent procedures were followed for all participants, as outlined by the institutional review board, prior to initiating any study procedures. Additionally, fidelity of implementation of the SCI-A program was measured throughout again by SCI-A staff who were knowledgeable of, but not directly involved in, delivering the SCI-A program.

### 2.6. Direct Observation and Reliability

#### 2.6.1. Target Measures

Each observation of in situ social behavior was for 10 minutes. Target measures were collected via the Multi-Option Observation System for Experimental Studies (MOOSES) [35, 36], software designed specifically for recording behavioral data. MOOSES automatically provided an output measure of both frequency and duration of each target behavior. 

For all participants, data were collected on appropriate and inappropriate initiations, responses, and continuations directed towards peers and to adults. Inappropriate initiations, responses, and continuations occurred at such low rates that only appropriate initiations, responses, and continuations are reported in this study. *Initiations* were defined as any motor or vocal behavior directed to a peer or adult that attempted to occasion a response, including greeting, asking and answering questions, commenting, sharing materials, helping behavior, saying someone's name, and gesturing to an item while looking at a peer. Initiations were required to be relevant to the context, socially appropriate, with no other conversation taking place prior to the initiation (appropriate interruptions to enter a conversation was an exception). *Responses *were defined as any motor or vocal behavior directed to a peer or adult that acknowledged an initiation within 5 seconds (e.g., looking when name was called, following directions or request, answering a question, and nodding head). Finally, *continuations *were defined as response directed to a peer or adult that maintained an ongoing interaction (i.e., follows the response of another peer or adult or participants own response within 5 seconds).

#### 2.6.2. Reliability

In order to obtain interobserver agreement (IOA) a second observer was present for 22 (24.4%) of the study's 90 observation sessions. The mean rate of agreement for all 22 sessions was 82%. Secondly, MOOSES provides a Cohen's Kappa coefficient and the mean score for the total IOA sessions was  .66. Kappa is always less than or equal to 1. A value of 1 represents perfect agreement and values less than 1 represent less than perfect agreement therefore, a kappa coefficient of  .66 represents substantial agreement [37].

#### 2.6.3. Fidelity

During SCI-A delivery fidelity was coded on 100% of all 31 sessions by SCI-A staff who were present during the intervention implementation. Across all lessons, mean fidelity scores indicated acceptable high fidelity within each of the four coded domains: content: 90%; process: 92%; behavior: 97%; feedback: 78%. Interobserver agreement was collected on 33% of all sessions and calculated by dividing the total number of agreements by the total number of agreements plus disagreements and multiplying by 100%. IOA for each process code was the following: content: 90.4%; process: IOA = 90.2%; behavior: M = IOA = 83.3%; feedback: M = 74.0%.

## 3. Results

Participant scores on assessments prior to beginning SCI-A and after SCI-A are presented in Table 3. The percentage of change in participants' scores from pre- to postintervention is indicated. Paired samples *t*-tests were conducted on the pre- and postintervention scores to determine if differences occurred. It should be noted that given the small sample size the significance of the statistics below is questionable and should be viewed as an initial investigation of the use of the SCI-A program in a school setting.  Table 4 reports the mean level of initiations, responses, and continuation in baseline and during the SCI-A program in the lunch setting for all six participants. The mean level of initiations, responses, and continuations in baseline and during the SCI-A program was available for three participants in the math setting and these data are reported in Table 4. 

### 3.1. Social Abilities

One general education teacher, who was blind to the details of the SCI-A program, completed the SRS, pre- and postassesment per participant. The SRS was intended to provide a general measure of change in social competence for individuals participating in the SCI-A program. The SRS total score serves as an index of severity of social deficits across the autism spectrum. The combined group showed an improvement on the total SRS score (Δ = 28%, *t* = 2.03, *P* < .10). Six participants made positive improvements on the total SRS score from pre- to postassesment. In addition to the total score, the SRS provides a score for five subscales including social awareness, social cognition, social communication, social motivation, and autistic mannerisms. For the combined group improvements were found on the social communication subscale (Δ = 29.0%, *t* = 2.20, *P* < .10) and the social motivation subscale (Δ = 31%, *t* = 2.82, *P* < .05). In addition, four of the participants made positive gains on all five of the SRS subscales.

### 3.2. Theory of Mind

The Theory of Mind (ToM) tasks and the Faux Pas stories measure changes in theory of mind and perspective taking. For the ToM tasks, all participants passed the Candy Box test in both pre- and postassessment. For the Sally-Anne task, two participants failed in pre- but then passed on the postassessment while the other participant passed in pre- but failed in postassessment. All participants passed the Friends ABC Story in both pre- and postassessments, except one participant who failed during pre- but passed during postassesment. Finally, four participants passed the Ice Cream Story in both pre-and postassesment yet, Jason and William both failed in pre- but passed in postassessments. In summary, the results indicate somewhat mixed results however this is congruent with previous studies using ToM tasks with the HFA/AS population [17, 38, 39].

### 3.3. Emotion Recognition

The DANVA 2-CF and the Reading the Minds in the Eyes test provide a measure of change from pre- to postintervention in the ability to recognize facial expressions and to interpret emotional and mental states. For the DANVA, the combined group showed an improvement from pre- to postassessment (Δ = 10%, *t* = 3.05, *P* < .05). Five participants showed an improvement on the ability to recognize facial expressions and to interpret emotional and mental states. The percent changes from pre- to postassessment for the Reading the Mind in the Eyes test and the Faux Pas Stories for the combined group were not significant. Two participants showed a positive change from pre- to postassessment on the Reading the Mind in the Eyes and four participants showed an improvement on the ability to recognize the social faux pas that were portrayed in the vignettes.

### 3.4. Executive Functioning

The BRIEF is included in the SCI-A assessment battery to provide a measure of change in executive functioning. The BRIEF was completed pre- and postassesment per participant by the same general education teacher who completed the SRS. The BRIEF is made up of two subscales that are combined to create the Global Executive Composite. According to teacher report, there was an improvement on the Global Executive Composite (Δ = 10%, *t* = 2.29, *P* < .10) as well as the Metacognition Index (Δ = 11%, *t* = 2.70, *P* < .05). Four participants made positive gains on the Behavioral Rating Index however the group mean score for this index from pre- to postintervention was not significant.

### 3.5. Generalization Results

Data for all six participants who were monitored during lunch suggest that generalization may have occurred beyond the SCI-A intervention setting. For each participant, a total social interaction (TSI) percentage was calculated in addition to initiations, responses, and continuations (IRC) for each generalization setting. TSI is defined by the combination of all appropriate and inappropriate IRC behavior (including IRC to peers and adults) within a 10-minute coding session. TSI was calculated by adding the duration in seconds of all social interaction within a 10-coding session. As noted previously, separate rates for inappropriate IRC were not provided as these behaviors occurred at very low rates.  Table 4 shows that all participants, with the exception of Ryan, made increases in TSI from baseline to intervention. The three participants for whom data was available in the math setting all made positive gains as well in TSI from baseline to intervention and these data are presented in Table 4. 

## 4. Discussion

The preliminary results reported here indicate that the SCI-A curriculum has potential in increasing social competence for adolescent with high-functioning ASD in a school setting. Moreover, the direct observation data provide evidence that the SCI-A program may also have potential in positive changes in social competence within additional school environments beyond the intervention setting.

### 4.1. SCI-A in a School Setting

The results for the SCI-A assessment battery in the current study are similar to findings from other studies using interventions that are based on cognitive behavioral interventions for individuals with HFA/AS [15–17, 40]. Given the insufficient evidence-based social interventions for individuals with HFA/AS, the findings from the SCI-A assessment battery are particularly promising as part of an ongoing multiphase research plan [18]. The results reported here provide initial evidence that the SCI-A curriculum can be successfully implemented in a school setting with meaningful outcomes for adolescents with HFA/AS. The results also contribute to the growing evidence for using cognitive behavioral-based interventions [10] to address the social and behavioral deficits of individuals with HFA/AS [15–17, 41]. 

Although the measures used within the SCI-A battery have been used not only in repeated CBI-based social competence literature, as well as in national clinical research trials, debate remains surrounding the appropriateness of some of these assessments. One notable and consistent example of this can be found within the variability of response among the ToM tasks. There is ongoing debate in the literature regarding the term Theory of Mind, and if it too narrowly describes a larger construct of social perspective taking, as well as the utility of the most commonly used ToM measures. Some authors have found ToM tasks to be a reliable instrument [42], yet others have noted problems with these assessments, particularly when used in applied research, and in particular for those considered high functioning [43, 44]. Baron-Cohen et al. [33] caution the interpretation of ToM tasks and note that children may fail such tests for a variety of reasons, most notably that reading comprehension problems may interfere with success on these tasks. This is relevant for individuals with HFA/AS as some literature suggest reading comprehension difficulties in this population [44]. Furthermore, according to Stichter and colleagues, the research has not provided sufficient validation of these measures as either screeners, or as pre- postassesment for growth. In light of the variable results found in this study and others, these results should be interpreted with caution.

### 4.2. Generalization of SCI-A

Inline with the secondary purpose of the study, the current results indicate promise for the potential of the SCI-A program to generalize to untrained settings. Evidence for some generalization was found in the current study in the math and lunch settings for all participants as measured by their positive change in overall social interaction. Unique to this study is the ability to explore generalization as captured not only by the total social interaction (TSI) measure, but also specific areas of social interaction (initiations, responses, and continuations). This multifaceted approach to measuring social behavior is particularly relevant for individuals with ASD in that each individual presents distinctive social competence deficits and as was evident in the current study, intervention resulted in unique outcomes for each participant. 

For Shawn, from baseline to intervention, the most notable change in the lunch setting was his continuations to adults and to typically developing peers. It was observed in baseline and throughout the study that Shawn was a very shy individual and relied on others to initiate social interaction. Shawn's increase in continuations indicates that once an adult or peer initiated with him, he was attempting to maintain the conversation more so than prior to intervention. Shawn's reduced rates of behavior change in the math setting can be attributed somewhat to the setting. Though social interaction was encouraged during small group activities, it was observed that if a participant was working diligently on the assignment the math teacher rarely prompted the participants to work with others. During direct observation sessions, Shawn was often observed independently completing his math work and not engaging in social interaction. 

Jason's data from baseline to intervention indicates that he did increase his social interaction in the lunch setting, and as expected, the primary change was with adults as his baseline rates with peers were already fairly high. It was observed throughout the study that Jason exhibited very high rates of the target behaviors and unique to other participants in this study, teachers targeted decreases in several areas of social communication as a goal for Jason while in the SCI-A program. At times Jason would engage in inappropriate social behavior such as self-talk for long periods of time in which his conversation seemed to have no audience. During lunch Jason often sat next to the special education teacher who would occasionally comment or respond to Jason's self-talk, possibly perpetuating this behavior. Jason, like numerous individuals with HFA/AS, has a remarkable verbal fluency [2], however, he lacked the understanding of social cues that indicated when he was talking too much. The SCI-A program may not have impacted Jason's social behavior as much as desired in the lunch setting due to the long reinforcement history [45] and proximity to the special education teacher, therefore making this behavior more resistant to the SCI-A strategies. However, from baseline to intervention in the math setting Jason demonstrated positive changes with a decrease in IRC behavior toward peers, which can be interpreted as an improvement in turn-taking skills for this participant. The SCI-A program is designed to promote self-monitoring and awareness and perspective taking [17]; therefore the decrease in IRC toward peers may have been Jason's increasing awareness of sharing the conversational space with others. 

William maintained a consistent level of total social interaction from baseline to intervention in the lunch setting. The positive outcome for this participant was evident in the shift in the type of interaction he was having from baseline to intervention. Specifically, he reduced his rate of initiations and increased his responses and continuations to adults resulting in appropriate reciprocal conversations. Similar to Jason, William exhibited a high level of verbal fluency and also engaged in self-talk behavior. However, his special education teacher referred William in part because he tended to focus his verbalizations on repetitive and stereotypic topics and interests. Again, the special education teacher was very familiar with William's interests and would often comment on his self-talk, therefore possibly maintaining the conversation. However, William's increase in responses and continuations during intervention suggest that he was using appropriate conversational skills highlighted in the SCI-A curriculum such as turn-taking and sharing ideas in conversation. In the math setting, although William showed an increase in all IRC behavior to adults and peers the most substantial change was in his interactions with adults. During intervention William was maintaining his conversations with adults in the math setting more so than in baseline. 

During baseline observations Ryan demonstrated levels of social interaction that were comparable to his general education peers. However, Ryan often exhibited inappropriate conversation skills with his peers during conversation. Ryan showed an increase in initiations, responses, and continuations from baseline to intervention. Moreover, it was observed that Ryan decreased his inappropriate conversation skills almost completely. He showed the most growth in his ability to independently initiate an interaction with his peers as well as respond to social bids from others. Conversation skills are a core component of the SCI-A program with a specific focus on turn-taking in conversation as well as understanding the roles of a good speaker and a good listener [17]. It was clear that Ryan wanted to be social and made a number of attempts to be social with his peers during baseline however it appeared that he lacked the knowledge of exactly how to execute an interaction successfully. The SCI-A program therefore provided the necessary tools for this success. 

Chris exhibited appropriate levels of social interaction during baseline observations. From baseline to intervention Chris demonstrated an increase in his initiations, responses, and continuations to peers. The most substantial increase in social behavior was his level of continuations to peers. Again, continuations indicate that this participant was able to engage in more reciprocal social interaction during the SCI-A program as compared to baseline. In addition to Chris' changes in initiations, responses, and continuations, it was also observed that Chris showed changes in the type of interactions that he had with individuals with whom he was less familiar. Throughout the study it was noted that Chris primarily interacted with a small group of peers and if other peers or adults with whom he was not comfortable attempted to engage in conversation with him he would typically ignore these initiations or respond in a rude manner. As Chris moved through the SCI-A curriculum, it was reported anecdotally from teachers that he was more tolerant of others and was more likely to demonstrate appropriate social behavior with individuals outside of his group of familiar peers. 

Jeremy demonstrated relatively low rates of social behavior during baseline observations. He would attempt to initiate conversation with peers however he had difficulty maintaining conversations for any length of time. From baseline to intervention, Jeremy made improvements in his initiations, responses, and continuations. However, he made the most gains in his responses and continuations indicating a substantial improvement in this ability to maintain a conversation with this peers. Often Jeremy was observed initiating topics of conversation that were overtly academic and often uninteresting to his peers. The positive changes in Jeremy's social behavior may be attributed to his use of skills learned in the SCI-A curriculum that enabled him to accurately interpret the social cues provided by his peers. For example understanding facial expressions could have helped Jeremy realize when peers began to get bored with a conversation or skills from sharing ideas could have influenced his willingness to share the conversation space with his peers. 

The results discussed above indicate that there is evidence for some generalization of skills from the SCI-A program to the lunch and the math settings. The SCI-A program consists of a number of curricular components that are consistent with suggestions from the generalization literature to promote generalized skill use and in part explains the results found in the current study. Stokes and Osnes [46] provide principles and tactics for generalization programming; these include (1) taking advantage of natural communities of reinforcement, (2) training diversely, and (3) incorporating functional mediators. First, taking advantage of natural communities of reinforcement refers to using elements of the natural environment that already function to maintain the target behavior [47]. The SCI-A program is designed to promote relevant behaviors to address social competence deficits for individuals with HFA/AS. Specifically, the SCI-A program teaches appropriate conversation skills, to enable participants to interact successfully with their peers and therefore contact natural communities of reinforcement. Second, training diversely refers to maintaining the minimal level of training control possible while still producing behavior change [46, 48]. The SCI-A program utilizes a unique scaffolding of curricular constructs, meaning the program provides a process for the acquisition of skills sets in combination with opportunities to practice these skills over time with multiple partners in multiple role-playing scenarios [17]. Therefore as participants progress through the curriculum they are provided more complex skill sets and afforded more and more opportunities to practice resulting in higher levels of fluency of skills as they complete the program. Finally, incorporating functional mediators refers to taking advantage of relevant discriminative stimuli in the training environment that can be transferred to other environments to promote generalizations [46, 48]. An integral component of the SCI-A curriculum is within a foundation of applied behavior analysis; the teaching of cognitive strategies that are intended to result in self-monitoring and self-management processes that participants can recall in social situations to aid in appropriate social responding across multiple environments and multiple social partners.

### 4.3. Limitations

Limitations of the current study include the ongoing challenge in identifying and using appropriate and valid ToM measures to capture growth in this domain for the particular subset of participants studied in this project. To this end, standard measures used in previous studies were accessed to stay consistent with the desire to replicate and extend in the area of setting and generalization without additional new variables. A small number of participants is also a limitation of the current study. A small sample size is indicative of a pilot study; however this greatly impacts the generalizability of the finding. Additionally constraints manifested that were indicative of measuring social interaction in applied settings. These included not being able to control for variable environmental stimuli in each measured setting. For example, although the math class was designed to be a cooperative learning environment, it was not consistently delivered by the teacher in this manner. The lunch setting included multiple conversation partners and included a special education teacher that on occasion created her own social stimulus. For example, although the math class was designed to be a cooperative learning environment, it was not consistently delivered by the teacher in this manner. The lunch setting included varied conversational partners and included a special education teacher that on occasion created her own social stimulus. An additional limitation is that both the individuals who conducted the direct observations, as well as the coders for the fidelity of the SCI-A program, were knowledgeable of the study and therefore could have introduced their own bias into the data. Also, due to study constraints and the end of the school year it was not possible to investigate maintenance of changes over time. Finally, due to limited research staff it was not possible to investigate generalization of the SCI-A program to other school environments such as recess, free time, and to community and home settings. 

In summary, the present investigation replicated and extended previous research demonstrating the efficacy of a social competence intervention based on a cognitive behavioral framework. More importantly, this study showed that social competence skills acquired in a school-based social competence intervention may have the potential to generalize to multiple school environments. These findings are particularly relevant in light of the current critique regarding a lack of generalized outcomes for social and behavioral interventions in the field of autism spectrum disorders as well as the overwhelming need for evidence-based practices for adolescents with HFA/AS. However the current study was designed as an initial investigation and therefore all findings should be interpreted with caution. Future research should continue the validation of the SCI-A program by pursuing the next phases of research suggested by Smith and colleagues [18] including an evaluation of the efficacy of SCI-A in large-scale randomized clinical trials as well as community effectiveness studies to assess whether practitioners across multiple sites can implement the program with fidelity. Ongoing measures of generalization and maintenance will undoubtedly be an integral part of the next steps of this particular line of research and related areas of investigation in the area of social competence and for those with HFA/ASD.

## Figures and Tables

**Table 1:** Participant demographics.

Name	Age	Grade	% Time in Gen Ed	FS IQ score	ADI score	ADI sign.	ADOS score	ADOS sign.
Shawn	13	7	76	99	RSI = 18	Yes	Comm = 5	Yes (Autism)
					Comm = 14		RSI = 11	
					Beh = 2		Total = 16	

Jason	13	7	76	84	RSI = 22	Yes	Comm = 3	Yes (Autism)
					Comm = 14		RSI = 10	
					Beh = 8		Total = 13	

William	13	7	70	106	RSI = 3	No	Comm = 4	Yes (Autism)
					Comm = 8		RSI = 7	
					Beh = 2		Total = 11	

Ryan	13	7	100	111	RSI = 2	No	Comm = 1	Yes (Autism)
					Comm = 5		RSI = 7	
					Beh = 0		Total = 8	

Chris	12	7	70	91	RSI = 20	Yes	Comm = 5	Yes (Autism)
					Comm = 23		RSI = 10	
					Beh = 6		Total = 15	

Jeremy	12	6	72	129	RSI = 19	Yes	Comm = 5	Yes (Autism)
					Comm = 14		RSI = 9	
					Beh = 3		Total = 14	

Notes: FS: full scale IQ; RSI: reciprocal social interaction; Comm: communication and language; Beh: restricted and repetitive, stereotyped interest and behaviors; Sign: significance.

**Table 2:** Participant baseline social characteristics.

Name	Baseline social characteristics
Shawn	(i) Limited social interaction with peers
	(ii) Social interaction primarily targeted towards adults
	(iii) Difficulty initiating and maintaining conversation

Jason	(i) High rates of inappropriate social behavior (e.g., talking too loud, interrupting, dominating conversation)
	(ii) Repetitive and stereotypic topics and interests
	(iii) Engaged in “self-talk” (e.g., continued conversation with no audience)

William	(i) Limited social interaction with peers
	(ii) Social interaction primarily targeted towards adults
	(iii) Engaged in “self-talk” (e.g., continued conversation with no audience)
	(iv) Repetitive and stereotypic topics and interests

Ryan	(i) Social interaction levels comparable to general education peers
	(ii) High rates of inappropriate social behavior (e.g., talking too loud, interrupting, dominating conversation, aggressive tone)

Chris	(i) Social interaction levels comparable to general education peers
	(ii) Limited social interaction (except for a select group of peers and adults)

Jeremy	(i) Limited social interaction with peers
	(ii) Social interaction primarily geared towards adults
	(iii) Pedantic conversation topics
	(iv) Difficulty initiating and maintaining conversation

**Table 3:** SCI-A assessment battery: percent change from pre- to postassesment (*n* = 6).

	Individual change pre- to postassesment						Group level	
	Shawn	Jason	William	Ryan	Chris	Jeremy	Mean % Δ	*t*-value
*Student performance data*								
ToM: Candy Box	P/P	P/P	P/P	P/P	P/P	P/P		
ToM: Sally-Anne	P/P	P/P	F/P	P/P	F/P	P/P		
ToM: Friends ABC Story	F/P	P/P	P/P	P/P	P/P	P/P		
ToM: Ice Cream Story	P/P	F/P	F/P	P/P	P/P	P/P		
Reading in Mind's Eye	0	11	0	−19	50	0	7	0.42
Faux Pas Stories	−11	29	−10	11	12	12	7	1.00
DANVA	0	5	5	10	20	24	10	3.05*

*Teacher reports*								
SRS total	46	46	54	4	−30	48	28	2.03^+^
Social awareness	67	20	38	−9	−17	27	21	1.66
Social cognition	43	50	69	0	−57	58	27	1.34
Social communication	48	44	51	11	−35	54	29	2.20^+^
Social motivation	37	53	50	21	−25	50	31	2.82*
Autistic mannerisms	33	54	61	−11	−13	38	27	1.48
BRIEF global executive composite	18	7	20	−4	0	21	10	2.29^+^
Behavioral regulation	8	4	23	−4	−4	25	9	1.51
Metacognition	21	8	18	−2	1	17	11	2.70*

Notes: ToM measures P: pass, F: fail, indicated as pre/postassesment; Mind's Eye, Faux Pas, DANVA percent change calculated as (post-pre)/preassesment; SRS and BRIEF percent change calculated as (pre-post)/preassesment.

**Table 4:** Mean percentage of 10 min sessions engaged in IRC in lunch and math.

		Lunch		Math	
Subject	Dependent variable	Baseline	Intervention	Baseline	Intervention
Shawn	Initiation to peer	.2 (0–.5)	1.38 (0–4.8)	0	1.5 (0–10.8)
	Response to peer	.2 (0–.5)	6.4 (1.5–11.6)	0	1.8 (0–7.5)
	Continuation to peer	1.95 (0–4.5)	12 (1–35.8)	0	2.8 (0–14.3)
	Initiation to adult	1.86 (.6–3.4)	1.14 (0–4)	.29 (0–.89)	.8 (0–5.16)
	Response to adult	1.02 (0–2.3)	1.4 (0–3.16)	1.7 (1.2–3.9)	1.7 (0–3.5)
	Continuation to adult	1.04 (0–1.9)	2.5 (0–6.6)	2.1 (1.3–4.98)	1.0 (0–3.7)
	Total social interaction	9.1 (1–13.2)	24.9 (10.5–49.7)	3.9 (0–9.8)	9.7 (1–30.8)

Jason	Initiation to peer	7.3 (2.17–12.2)	10.9 (3.5–17.3)	11.6 (9.5–14.3)	9.1 (1.83–32.17)
	Response to peer	5.9 (2.3–7.3)	4.5 (1.3–9.8)	11.2 (6.83–18.16)	5.8 (2.83–8.83)
	Continuation to peer	22.7 (1–46.5)	10.5 (0–32.2)	31.2 (27.5–35.16)	12.4 (0–32.5)
	Initiation to adult	3.8 (0–9.8)	5.9 (2.3–10.3)	.61 (.16–.83)	1.8 (0–4.83)
	Response to adult	7.4 (0–19.5)	6.79 (2.2–10.2)	.9 (0–1.16)	3.7 (0–9.5)
	Continuation to adult	12.67 (0–30.3)	22.7 (10.3–43.2)	1 (0–2.3)	4.9 (0–14.33)
	Total social interaction	67.7 (59.3–74.6)	73.0 (54.3–86.2)	61.6 (51.6–75.3)	43.9 (14.8–87)

William	Initiation to peer	1.9 (1.3–2.5)	.9 (0–2.5)	.8 (0–1.5)	2.2 (0–4.6)
	Response to peer	3.9 (0–8.2)	2.2 (0–8.6)	.8 (0–1.6)	1.9 (0–5.3)
	Continuation to peer	9 (0–18)	7.3 (0–52.6)	.8 (0–1.5)	1.0 (0–3.6)
	Initiation to adult	6.61 (0–12.6)	3.9 (0–6.5)	1.5 (1–1.9)	1.5 (0–5.5)
	Response to adult	4.4 (0–7.5)	7.3 (0–12.5)	.83 (0–1.6)	4.6 (1.2–9.6)
	Continuation to adult	7.3 (0–14)	18.4 (0–19.2)	0	9.9 (0–32.5)
	Total social interaction	41.3 (28.1–60.6)	41.7 (19.6–85.6)	3.83 (0–9)	23.9 (11.5–23)

Ryan	Initiation to peer	2.3 (1–4.3)	8.9 (2.7–14)		
	Response to peer	4.8 (1.3–9.5)	13.4 (.83–19)		
	Continuation to peer	18.6 (5–61.3)	21.5 (0–31.6)		
	Total social interaction	54.6 (34.8–78.5)	47.3 (3.0–61.7)		

Chris	Initiation to peer	9.72 (8.8–10.6)	11.6 (4.8–20.6)		
	Response to peer	7.2 (5.8–9.5)	10.4 (4.6–15.8)		
	Continuation to peer	12.8 (2.8–28)	19.7 (0–30.7)		
	Total social interaction	31.2 (21.0–49.2)	41.8 (11.8–88.8)		

Jeremy	Initiation to peer	5.5 (0–16.5)	8.65 (1–19.3)		
	Response to peer	.66 (0–1.9)	12.65 (6.8–18.5)		
	Continuation to peer	6.78 (0–16.5)	23.9 (7–57)		
	Total social interaction	23.1 (1.7–46.3)	48.7 (34.8–76.8)		
